# The MRI spectrum of congenital cytomegalovirus infection

**DOI:** 10.1002/pd.5591

**Published:** 2020-01-06

**Authors:** Mariana C. Diogo, Sarah Glatter, Julia Binder, Herbert Kiss, Daniela Prayer

**Affiliations:** ^1^ Department of Image Guided Therapy University Clinic for Neuroradiology and Musculoskeletal Radiology, Medical University of Vienna Vienna Austria; ^2^ Department of Pediatrics and Adolescent Medicine Medical University of Vienna Vienna Austria; ^3^ Department of Obstetrics and Gynecology Medical University of Vienna Vienna Austria

## Abstract

Human cytomegalovirus (CMV) is an ubiquitous pathogen, with a high worldwide seroprevalence. When acquired in the prenatal period, congenital CMV (cCMV) is a major cause of neurodevelopmental sequelae and hearing loss. cCMV remains an underdiagnosed condition, with no systematic screening implemented in pregnancy or in the postnatal period. Therefore, imaging takes a prominent role in prenatal diagnosis of cCMV. With the prospect of new viable therapies, accurate and timely diagnosis becomes paramount, as well as identification of fetuses at risk for neurodevelopmental sequelae. Fetal magnetic resonance imaging (MRI) provides a complementary method to ultrasound (US) in fetal brain and body imaging. Anterior temporal lobe lesions are the most specific finding, and MRI is superior to US in their detection. Other findings such as ventriculomegaly, cortical malformations and calcifications, as well as hepatosplenomegaly, liver signal changes and abnormal effusions are unspecific. However, when seen in combination these should raise the suspicion of fetal infection, highlighting the need for a full fetal assessment. Still, some fetuses deemed normal on prenatal imaging are symptomatic at birth or develop delayed cCMV‐associated symptoms, leaving room for improvement of diagnostic tools. Advanced MR sequences may help in this field and in determining prognosis, but further studies are needed.

**What is already known about this topic?**

- Congenital cytomegalovirus (CMV) infection is a leading cause of congenital sensorial‐neural hearing loss and developmental delay.
- Congenital CMV remains underdiagnosed, partially due to lack of screening and awareness, but also due to unrecognized prenatal findings suspicious for CMV infection in prenatal imaging.
- Despite unspecific, ultrasound and magnetic resonance imaging (MRI) findings are essential in diagnosis and prognostication.

**What does this study add?**

- This study provides an overview of fetal brain and body MRI findings.
- It presents illustrative images and tables to facilitate performance and interpretations of fetal MRI in the context of congenital CMV.
- Application of advanced MR sequences in overcoming limitations of structural brain imaging.

**Abbreviations**
T2w: T2‐weighted
T1w: T1‐weighted
ADC: apparent diffusion coefficient
AF: amniotic fluid
CMV: cytomegalovirus
cCMV: congenital cytomegalovirus
DTI: diffusion tensor imaging
DWI: diffusion weighted imaging
EPI: echo planar imaging
FA: fractional anisotropy
FGR: fetal growth restriction
MRI: magnetic resonance imaging
MRS: magnetic resonance spectroscopy
mI: myo‐inositol
ssFSE: single shot fast spin echo
SSFP: steady state free precession
US: ultrasound
WM: white matter
WI: weighted images

## 1 INTRODUCTION

Human cytomegalovirus (CMV) is an ubiquitous pathogen, with a worldwide seroprevalence of 40%‐100% in the adult population.^1^, ^2^, ^3^ In immunocompetent individuals, CMV infection is often (80%‐95%) asymptomatic or associated with mild flu‐like symptoms.^2^, ^4^ However, when acquired during fetal development, congenital CMV (cCMV) is a major cause of neurodevelopmental deficits and the most common cause of nongenetic sensorineural hearing loss (SNHL).^5^ Around 10%‐20% of infants will be symptomatic at birth,^6^, ^7^, ^8^, ^9^, ^10^ and both symptomatic and asymptomatic newborns remain at risk of sequelae.^7^ Long‐term sequelae are expected in 40%‐60% of symptomatic survivors, and 10%‐20% of asymptomatic children.^7^, ^11^, ^12^, ^13^ This number may be much higher if longer follow up would be routinely available and accounted for lesser symptoms such as delayed cognitive, motor or language development, or behavioral issues, in some cases only detected at the start of school age.^14^

Despite extensive literature available, cCMV remains an underdiagnosed condition particularly in cases not associated with characteristic prenatal/neonatal findings or with late‐onset symptoms, delaying initiation of treatment. This is partially related to the lack of universal CMV screening of pregnant women, which is not recommended given the lack of proven effective therapy during gestation.^15^

Until recently there were no viable options to prevent maternal‐fetal CMV transmission or improve outcome of infected fetuses, leading prenatal imaging to focus on detecting existing central nervous system (CNS) anomalies associated with dismal developmental outcomes. With the introduction of in utero treatment options, identifying fetuses at risk for neurodevelopmental sequelae becomes more relevant, although one should acknowledge the fact that this treatment options are still of experimental nature and have potential side effects.^16^, ^17^ A significant percentage of fetuses deemed normal on prenatal imaging become symptomatic at birth or develop delayed cCMV‐associated symptoms, leaving room for improvement. This, together with increased awareness and the shifts in paradigms of transmission, screening and treatment a review on this topic was warrant, focusing on MRI and advances in imaging, and how these may contribute to counseling of cCMV patients.

## 2 METHODS

We identified published studies and review papers related to congenital CMV infection by searching the MEDLINE database for English‐language articles published from 1962 (first available article) through July 2019 using the keywords “cytomegalovirus” or “CMV” and “congenital.” References of the selected articles were further checked for papers that may have escaped the primary search. Priority was given to information published in higher impact journals and studies with clear methodology and high number of subjects over case reports, case series or convenience samples. All relevant literature used can be found in the reference section of this paper.

## 3 CMV INFECTION IN PREGNANCY

### 3.1 Infection

CMV is part of the *Herpesviridae* family. It is transmitted via smear infection, through direct contact of mucous surfaces with infectious body fluids,^17^, ^18^ establishing a lifelong latent infection in the host, with periodic reactivations which can be a source of disease.^19^ The prevalence of cCMV ranges from 2 to 20 per 1000 live births, depending on factors such as geography and socioeconomical status.^6^, ^20^, ^21^, ^22^, ^23^, ^24^

Several factors contribute to the burden of cCMV in childhood, both in terms of morbidity and mortality. These include the limited awareness of parents and healthcare professionals,^25^, ^26^, ^27^ underrecognition of infection (often asymptomatic in pregnant women and newborns), lack of established screening programs, delayed‐onset of sequelae (making retrospective diagnosis challenging), and the absence of vaccines and limited proven efficacy of current treatments.^16^, ^22^

Maternal infections are described as primary (no preconceptional immunity) vs nonprimary (preconceptional immunity; including reactivations and infections by a different strain). This division has been used to stratify the risk of fetal transmission as well as the severity of sequelae.^10^ However, the dogma that prior maternal immunity can protect against symptomatic cCMV infection has been challenged^10^, ^22^ by former^28^ as well as recent studies,^20^, ^21^, ^29^, ^30^, ^31^, ^32^, ^33^, ^34^, ^35^, ^36^, ^37^, ^38^ and the impact of quantitative preconceptional immunity in intrauterine CMV transmission rates remain undefined.^10^, ^22^ In some prospective studies the risk of long‐term neurodevelopmental sequelae has been shown to be comparable in primary and nonprimary infections.^30^, ^33^, ^34^, ^39^, ^40^, ^41^

A unique feature of cCMV is that its prevalence increases with an increase in CMV prevalence in the maternal population, without reaching a level at which cCMV incidence falls.^10^, ^42^, ^43^ As such, the high seropositivity in the antenatal maternal population means that a high percentage of congenitally affected babies are born to immune mothers.^44^

Timing of seroconversion/fetal infection is a strong predictor of postnatal sequelae^9^, ^12^, ^40^, ^45^, ^46^, ^47^ with periconceptional and early gestational associated with a higher risk of symptomatic disease at birth.^39^, ^48^ Populations at higher risk of fetal transmission throughout pregnancy include women with underlying immunity deficits primary or acquired.^10^, ^49^

cCMV is the most common nongenetic cause of SNHL, accounting for 25%‐30% of sensorineural hearing loss in childhood.^50^ Long‐term follow up studies have determined that 8%‐10% of patients will present neurodevelopmental abnormalities, independent of the presence of symptoms at birth.^51^, ^52^

Maternal or fetal screening is currently not recommended or performed, although a recent American study established the cost‐effectiveness of universal screening in pregnancy, granted CMV incidence is at least 0.82%, or even lower if behavioral interventions to prevent transmission and further pharmacological interventions are being offered.^15^ The current consensus is that serology tests (IgG, IgM, IgG avidity) should be offered to pregnant women who develop flu‐like symptoms not attributable to another specific infection, or when imaging findings (ultrasound [US] or magnetic resonance imaging [MRI]) are suggestive of cCMV.^16^ Intrauterine infection can be confirmed by polymerase chain reaction detection of CMV DNA in the amniotic fluid (AF) after 21 gestational weeks (GW) and at least 6 weeks after infection, as a sufficient time is required for placental crossing and for fetal renal function to become sufficient for viruria to be detectable.^9^

Patient education and institution of hygiene measures have an effectiveness of up to 85% in prevention of primary CMV.^53^, ^54^ If maternal infection cannot be prevented, the most widely spread treatment options are hyperimmune globulin 55, 56 and antiviral drugs (valacyclovir, valganciclovir) to reduce the likelihood of fetal transmission or ameliorate fetal sequelae,^55^, ^56^, ^57^, ^58^, ^59^, ^60^ although no randomized controlled trials have proven a definitive benefit. As such, treatment options should be discussed on an individual basis.

### 3.2 The role of MRI in congenital CMV

Imaging in cCMV has two main objectives: detection of fetal structural anomalies for correct diagnosis and provision of prognostic information.

In the absence of universal screening, detecting and raising the possibility of a fetal CMV infection is paramount. This raises the importance of AF or newborn testing, allowing fetal treatment in selected cases, and avoiding a lengthy and costly diagnostic journey for children with cCMV born with nonspecific symptoms (mean specificity of neonatal symptoms: 12%) or developed delayed symptoms.^5^ Further, an accurate diagnosis helps parents in future reproductive decision making, and reduce the stress and anxiety from an uncertain diagnosis.

While US is the method of choice for fetal imaging, MRI has an established added value in the detection of fetal brain anomalies in several conditions,^61^ and has a growing potential for the evaluation of structural but also functional as well as metabolic imaging methods.

Detection of cCMV‐related CNS anomalies is a predictor of poor outcome among infected fetuses,^62^ while a normal neurosonographic examination performed by expert sonographers is a good predictor of normal neurodevelopmental outcome.^63^, ^64^ MRI has also been used in multiple studies concerning CMV, with a high negative predictive value (96.8%‐99%) for neurological impairment and SNHL.^65^, ^66^

As such, familiarity with the typical findings is essential. Furthermore, there is sparse literature regarding body findings in cCMV on fetal MRI. Below we aim to summarize and illustrate the most relevant MRI findings.

### 3.3 Central nervous system MRI

Prenatal imaging in cCMV is primarily focused on the brain, given the neurotropism of the virus.^67^ On a cellular level, cCMV may infect an array of cell types, including neurons, astrocytes, radial glia and endothelial cells, and may result in a number of insults to neuronal proliferation, migration, and cortical cell organization.^67^, ^68^, ^69^, ^70^ From an imaging point of view, visible (macroscopic) damage is a late finding that can show variable and progressive (and some regressive) features on prenatal imaging.

MRI findings in cCMV are often unspecific, with ventriculomegaly and white matter (WM) signal abnormalities being the most commonly described CNS anomalies.^71^ More characteristic (but less frequently observed) features include temporal lobe lesions (abnormal WM, cysts, and enlargement of the temporal horns), ventriculitis and intracranial calcification.^66^, ^72^ A summary of the main findings and their MRI characteristics can be found in Table 1.

**Table 1:** List of possible magnetic resonance imaging (MRI) findings in fetuses with congenital cytomegalovirus (cCMV)

	Finding	MR characteristics	Notes
Brain	WM hyperintensities	T2‐hyperintense inhomogeneities of the WM Low SI on DWI Low SI on T1w‐/T2w FLAIR Use MRS?	Subjective Difficult to interpret particularly in the third trimester Temporal lobe worst prognosis.
	Ventriculomegaly	Increased lateral ventricle size (>10 mm), measured at the atria Mild: 10‐12 mm Moderate: 12‐15 mm Severe: >15 mm	May be uni‐ or bilateral Mild to moderate: low risk; severe: high risk of sequelae^73^
	Cysts/pseudocysts	Well defined lesions with SI similar to CSF on all sequences Most often periventricular	Inconsistent nomenclature temporal polar lesions highly predictive of CMV infection
	Ventriculitis	T1w and T2w hyperintensity of the ventricular rim. On T2WI not visible due to juxtaposition to CSF; T2w‐FLAIR useful if T1 is not informative	Rare finding. Most common lateral ventricles. Periventricular hyperechogenicity
	Intraventricular septations/adhesions	Tissue strands (T2w low SI) crossing the ventricles	Most common occipital horns
	Cortical malformations/polymicrogyria	Cortical infoldings located in abnormal positions; Thickened cortical ribbon Blurry gray/WM margins on T2WI/FLAIR	MRI superior to US
	Clefts (schizencephaly/porencephaly)	Schizencephaly: transmantle cleft, lined by T2 hypointense (=cortex) ribbon^81^ Porencephaly: cleft with no cortical lining. Margins may show high T2w/FLAIR hyperintensity	Lesions secondary to disruption. Final manifestation depends on time of insult.
	Calcifications	Low T2 and high T1 signal, often subtle Low T2*/EPI SI	Periventricular > deep gray nuclei > white matter
	Cerebellar hypoplasia/dysplasia	Small vermis and/or hemispheres Increased infra/retrocerebellar space (megacisterna magna >8 mm^73^) May have associated focal signal changes (ie hemorrhage, calcifications)	Rare fetal MRI Common postnatal imaging
	Hippocampal dysplasia	Dilated temporal horns Verticalization of the hippocampal± internal temporal lobe atrophy	Often not described in fetal MRI. Common postnatal imaging. (DeVries)
	Lenticullostriate vasculopathy	US diagnosis Low SI T2WI on basal ganglia Calcification (low EPI/T2* and high T1 SI) of basal ganglia	Late finding on MRI
Body	Hepato/Splenomegaly	Increased size of liver and/or spleen	Special attention should be payed to signal (easily missed on US)
	Liver	Low T1‐ and T2 SI may depict global liver involvement (fibrosis/insufficiency) May have high T2*/EPI SI	Intrahepatitic calcifications better identified on US
	Effusions (pericardial, pleural, ascitis)	Fluid collections in the pericardial, pleural or abdominal cavities. Identical signal to CSF/AF on all sequences.	Pulmonary hypoplasia may ensue secondary to pleural effusion or ascites^105^
	Skin edema	Increased thickness of skin + subcutaneous tissue High T2 SI, low T1 SI	
	Hyperechogenic bowel	No findings on MRI Increase T1w meconium signal if blood ingestion	US change. MRI normal if no associated anomalies^103^
Other	Placenta	Placentomegaly placental thickness (>40 mm) Inhomogeneity on T1/T2 May have T2*/EPI	
	Amniotic fluid	Oligo‐/Polyhydramnious High T1SI if intra‐amniotic hemorrhage	T2 low sensitivity to hemorrhage FLAIR may show false positive to hemorrhage (high SI) due to fetal movement

Abbreviations: AF, amniotic fluid; CSF, cerebral‐spinal fluid; IUGR, intrauterine growth restriction; SSFP, steady‐state free precession; WI, weighted images; WM, white matter.

**Figure 1:**
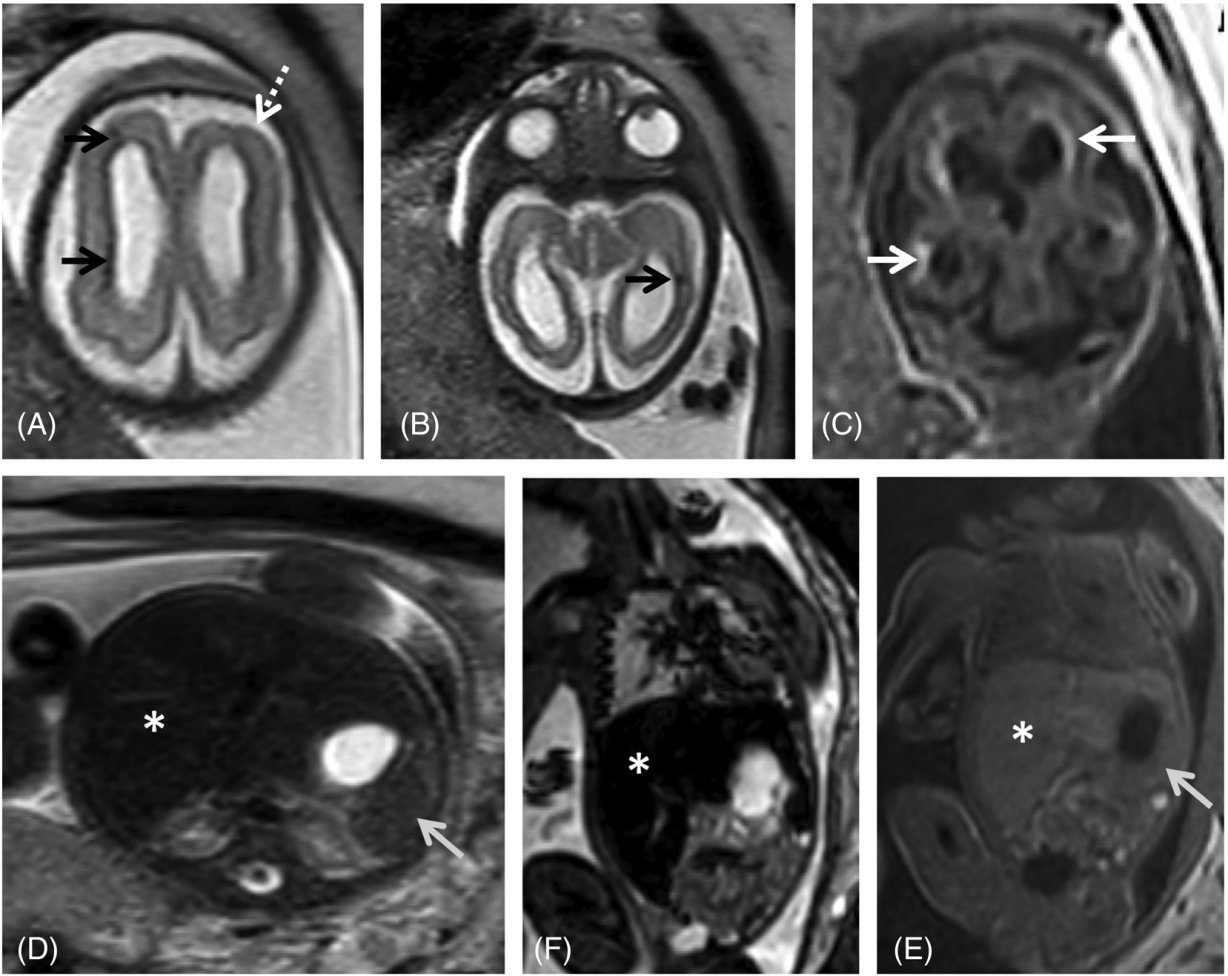
Fetal magnetic resonance imaging (MRI) at 29 gestational weeks referred for microcephaly and splenomegaly. There is parenchymal loss, with global thinning of the cerebral mantle and consequent bilateral ventriculomegaly. Small areas of focal signal anomaly can be detected on T2WI (A, B, black arrows), as well as frontal polymicrogyria (A, white dashed arrow). Calcifications can be identified on T1WI (c, white arrows). Enlarged spleen (gray arrow) and liver (asterisk) can be identified on T2W steady state free precession (D), echo planar imaging (E) and T1W images (F), with slight signal intensity anomaly on the latter

**Figure 2:**
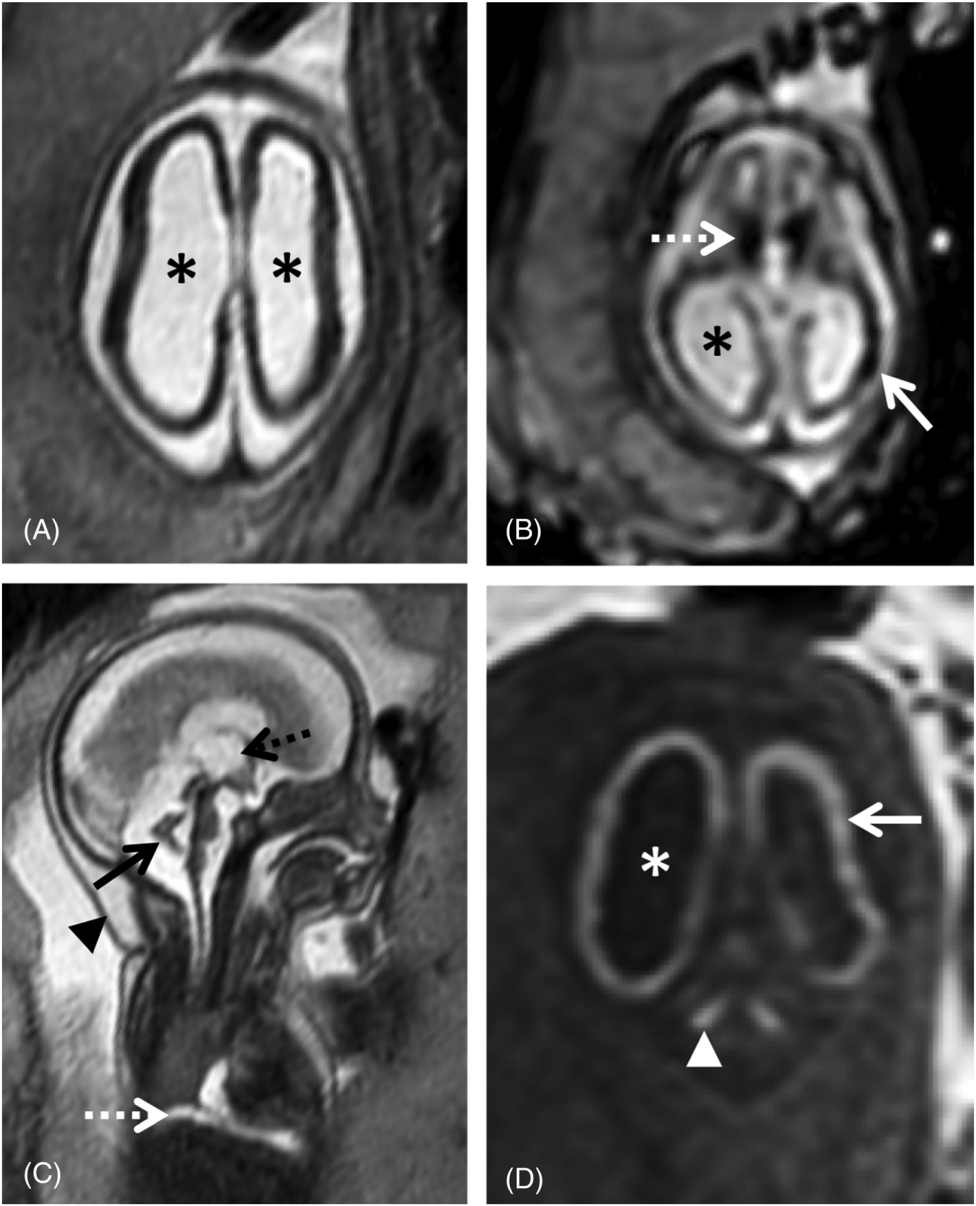
24 gestational weeks fetus referred for fetal magnetic resonance imaging (MRI) for suspected lissencephaly and cerebellar hypoplasia. T2w single shot fast spin echo axial (A) and sagittal (C), axial echo planar imaging (EPI) (B) and coronal T1WI (D) show a marked reduction of the cerebral parenchyma thickness, with diffuse low SI on T2WI (A) and severe ventriculomegaly (asterisk), including dilatation of the third ventricle (C, black dashed arrow). There is diffuse high SI on T1WI of the supratentorial parenchyma (D, white arrow) compatible with presence of calcifications, confirmed on EP images (B) and particularly evident in the basal ganglia (B, white dashed arrow). There is cerebellar hypoplasia (C, black arrow) associated with deep gray nuclei calcifications (D, white arrowhead). Furthermore, small pleural effusion can be seen on sagittal T2WI (C, white dashed arrow). Findings were confirmed on postmortem MRI (not shown). Additionally, there is skin edema/thickening of the skin (C, black arrow head)

Ventricular anomalies are often found in cCMV patients (Figure 1). Ventriculomegaly is mild to moderate (<15 mm) in most patients.^72^ Severe ventriculomegaly (>15 mm) on fetal MRI is associated with worse prognosis (Figure 2).^73^ Extreme forms of ventriculomegaly may mimic acqueductal stenosis‐related hydrocephalus, or less common presentations such as mega‐cisterna magna.^74^

**Figure 3:**
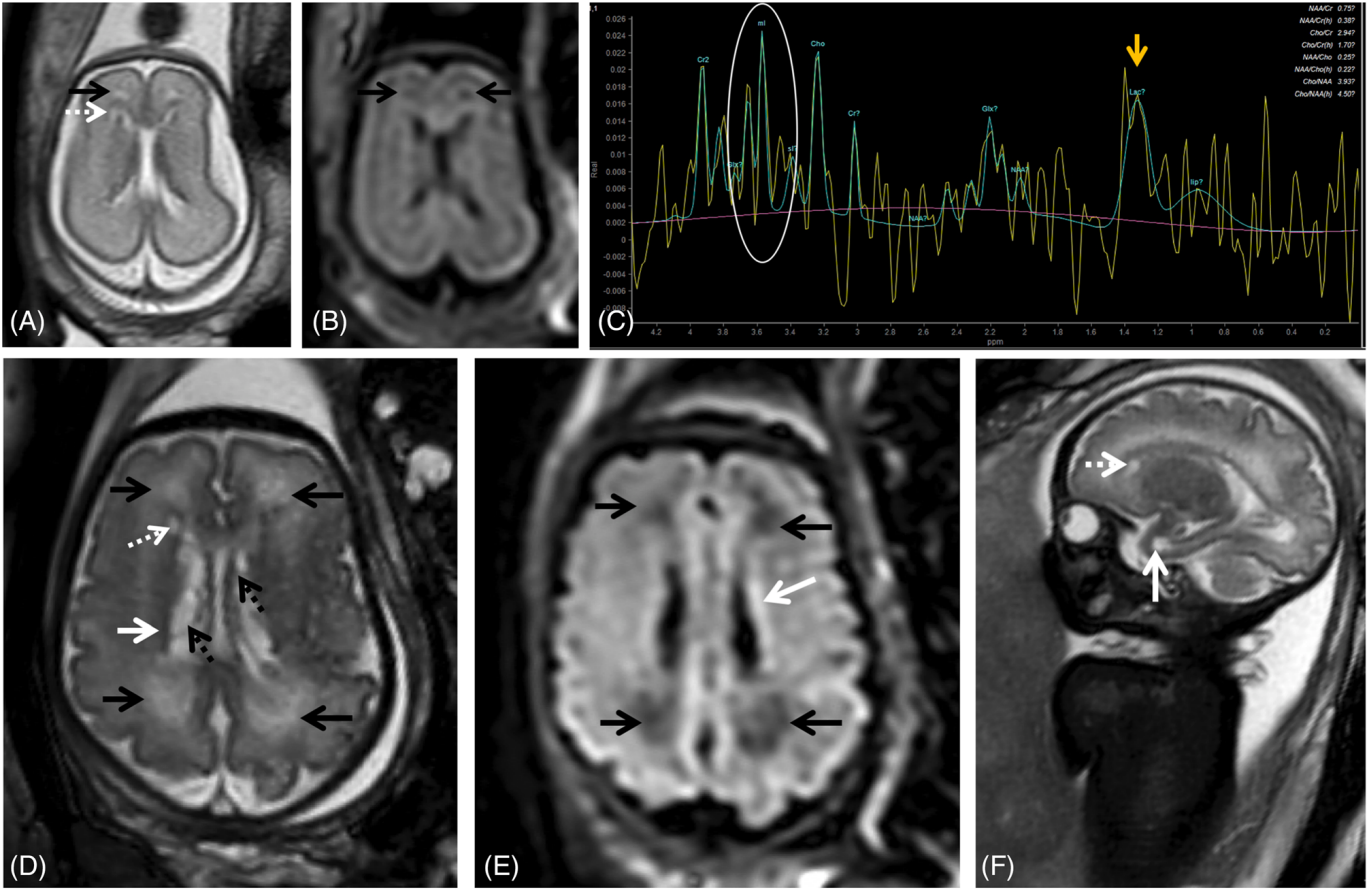
Fetal magnetic resonance imaging at 24 gestational weeks (GW) referred for maternal cytomegalovirus seroconversion and normal brain ultrasound. On T2 weighted images (WI) slight periventricular caps can be identified frontally (A, black arrow) as well as a small periventricular cyst. PRESS spectroscopy performed with a short TE (35 ms) depicts a myoinositol peak (myo‐inositol, white circle, C) and a lactate peak (Lac, yellow arrow, C), raising the suspicion of more extensive brain involvement. Follow up at 34 gestational weeks shows progression of white matter signal changes in the frontal and parieto‐occipital regions bilaterally (black arrows, D) that also have translation on the FLAIR image (black arrows, E). The presence of the small periventricular cyst can be confirmed (D, F, white dashed arrow) and there is the additional finding of a temporal pole cyst (F, white arrow) and irregularity and signal alteration of the ventricular lining (hypointense on T2WI, D, white arrow; and hyperintense on t2W FLAIR images, E, white arrow) with intraventricular septations/pseudocysts (D, black dashed arrow)

Ventricular septations/adhesions, also called pseudocysts, are another nonspecific finding.^72^, ^74^, ^75^ They are more common in the occipital horns and can be identified as thin strands of tissue crossing the ventricles on T2‐weighted images (Figure 3); longer echo times may be needed to detect these structures, and T2w FLAIR may improve visualization of intraventricular lesions.^76^, ^77^ Pseudocysts in the caudothalamic groove or in the lateral aspect of the frontal horns have also been described^66^ and all seem to carry a good prognosis when isolated.^66^, ^73^ Other cystic lesions particularly in the temporal pole region or when involving the WM are associated with neurological sequelae.^13^, ^65^

**Figure 4:**
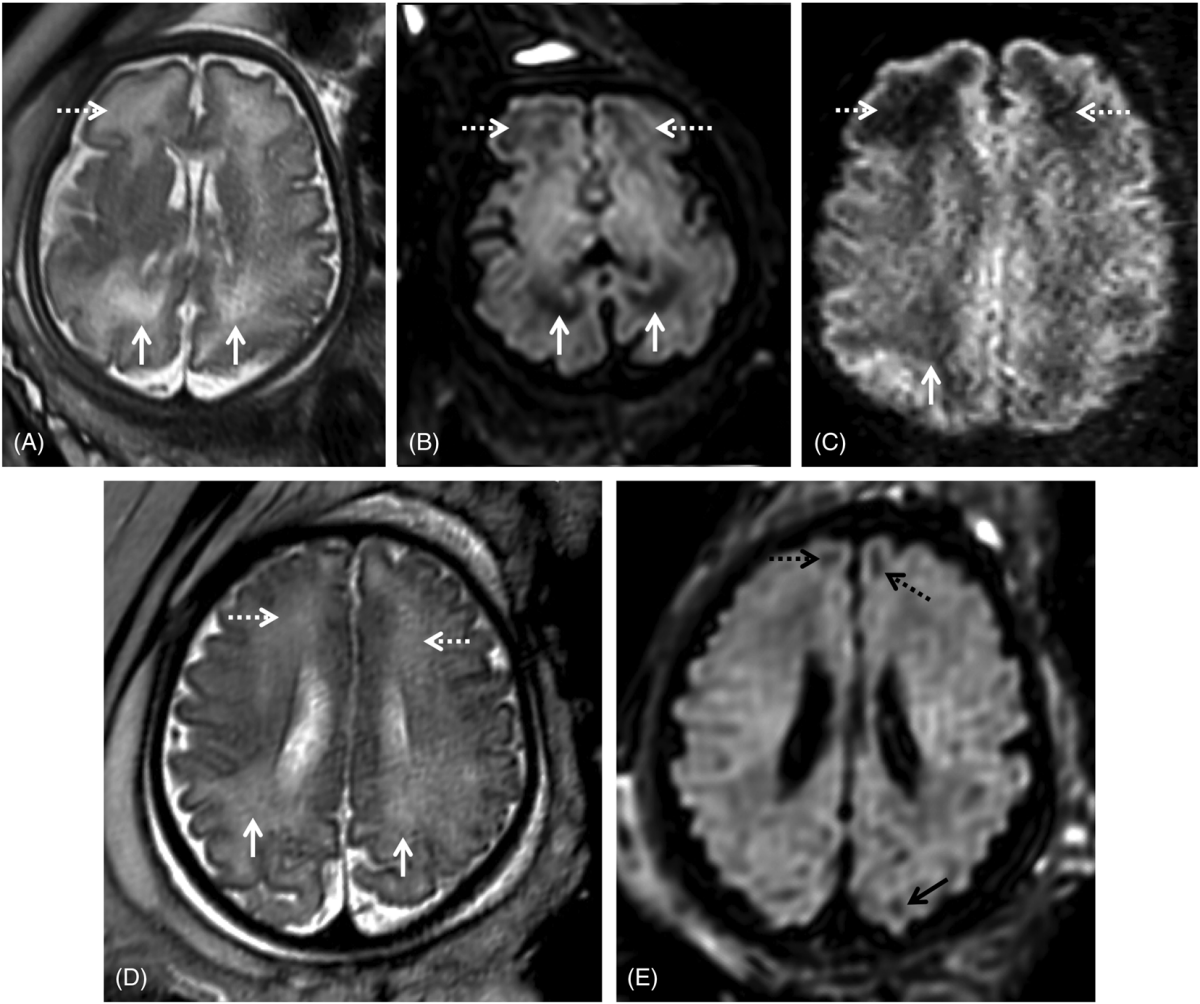
Two fetuses referred for fetal magnetic resonance imaging at 34 gestational weeks for congenital cytomegalovirus (cCMV) infection (A‐C) and abdominal cyst (D‐E). White matter hyperintensities can be identified on T2WI in the frontal (A, D, white dashed arrows) and parietal‐occipital parieto‐occipital (A, D, white arrows) regions. On T2w echo planar imaging‐FLAIR images (B, E) there is corresponding hypointensity in the cCMV patient (B, white/dashed arrows), as well as low SI on the zoom diffusion weighted image (C, white/dashed arrows), but not the control (E) except in the expected gyral crests corresponding to remnants of the subplate (E, black dashed and full arrows)

T2‐weighted (T2w) hyperintensities of the WM are a subjective diagnosis and difficult to interpret, particularly in the third trimester, when T2 inhomogeneities are often found in the normal brain (Figure 4, Supporting Information Figure S1). When interpreting possible cCMV cases, mild WM signal changes that would most likely be dismissed in other contexts can be overvalued in the fear of overlooking a potential CNS lesion. In most studies isolated WM T2 hyperintensities of the frontal and parieto‐occipital WM are associated with normal early development,^46^, ^63^, ^64^, ^78^ although these studies do not account for long term developmental outcome. In contrast, Lucignani et al found that WM alterations and ventriculomegaly predict an increased risk of adverse neurological outcome in congenitally CMV infected infants, symptomatic and asymptomatic at birth.^47^ WM changes in the temporal lobe are associated with a higher chance of neurological sequelae.^65^ Additional MR sequences may be used to help clarify these findings. Yanir et al suggested the use of diffusion‐weighted imaging (DWI) (Figure 3),^79^ and FLAIR imaging may help in depicting areas of edema or disruption of normal lamination (Figures 3 and 4).

**Figure 5:**
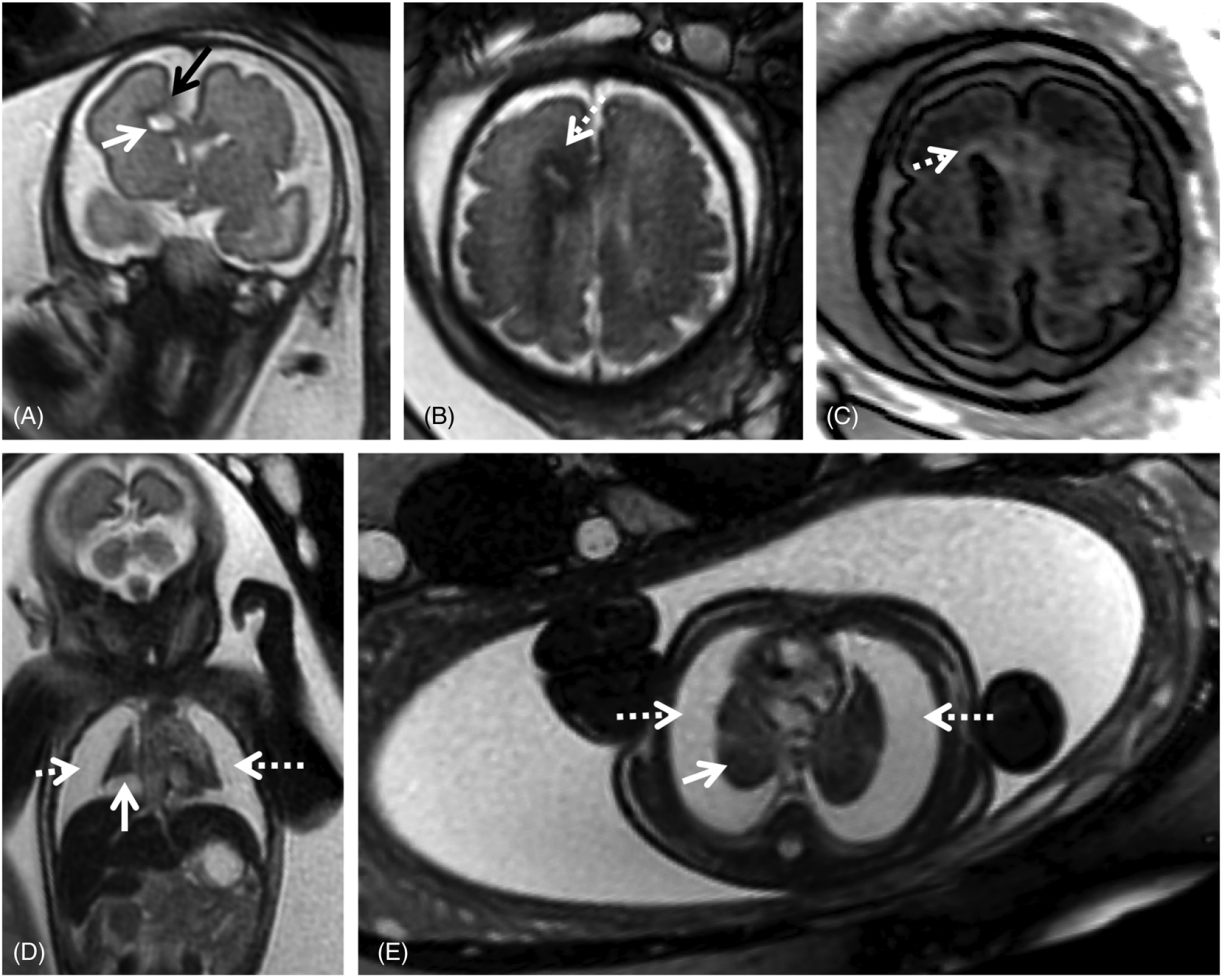
Magnetic resonance imaging of fetus referred at 31 gestational weeks for hydrothorax and suspected frontal brain lesion. On T2WI over the fetal brain (A,B) it is possible to identify a destructive lesion of the right frontal lobe, with focal parenchymal loss (A, black arrow), with associated intraparenchymal white matter cystic lesion (A, white arrow) and altered signal of the surrounding parenchyma, with T2 hypointensity (B, white dashed arrow) and high signal on T1‐weighted FLAIR imaging (C, white arrow), suggesting gliotic changes. There are extensive bilateral pleural effusions (D, E, dashed white arrows), with severe reduction of the lung volume (D, E, white arrows)

Fetal MRI is complementary and often superior to US in detecting abnormal gyration and myelination.^46^, ^66^, ^80^ Depending on the timing of infection and injury extent, a wide spectrum of abnormalities can be seen. cCMV affects the germinal matrix, leading to neuronal cell loss and diffuse disruptions of migration when the insult occurs before 16‐18 weeks of gestation. (Micro)lissencephaly; polymicrogyria can be seen in injuries that occur between 18 and 24 weeks whereas fetuses affected in the third trimester usually have a normal gyral pattern.^13^, ^72^, ^81^, ^82^ Polymicrogyria (particularly in the frontal and perisylvian regions) is the most commonly observed migrational abnormality,^83^ characterized by cortical infoldings located in abnormal positions, resulting in an appearance of too many infoldings for gestational age^80^; a thickened cortical ribbon with irregular gyration and possible blurry gray/WM margins may also be identified on T2WI (Figures 1 and 5).^80^ Vascular injury (by direct endothelial infection or placental involvement) at critical time points of development is associated with the fetal brain disruption sequence.^68^ Severe injury may also lead to clefts.^83^ Both schizencephaly and porencephaly appear as a transmantle cleft extending from the ventricular lining to the cerebral surface. The former is lined by a cortical ribbon, usually polymicrogyric,^80^ while the latter results from destructive changes occurring after the completion of neuronal migration and thus the cleft is not lined with gray matter.^84^

Calcifications are a classical finding in cCMV, typically described in US. Intracranial calcifications are more common in the periventricular region, but can also occur in the basal ganglia, as a manifestation of lenticulostriate vasculopathy, and in the brain parenchyma. They present as areas of low T2w and high T1w signal, and low signal in echo planar imaging (EPI) (Figures 1 and 2). Punctate calcifications may be difficult to detect on fetal MRI.^72^

The suspicion of cCMV infection should be raised when aforementioned lesions occur in the anterior temporal lobe, also termed “polar temporal lesions”.^66^, ^71^ There may be WM T2 hyperintensity (Figure 1) with or without swelling, cysts and/or pseudocysts, and mild dilation of the temporal horn,^81^ which may reflect hippocampal dysplasia reported on postnatal MRI examinations.^85^ Decreased volume of the temporal lobes compared with unaffected fetuses has also been described.^86^ These anterior temporal lobe features can be seen alone or, more often, in combination and are commonly bilateral. These findings are better evaluated with fetal MRI than with US.^66^, ^75^

Cerebellar anomalies are a common feature in postnatal cCMV MRI,^75^, ^81^ but are less common on fetal MRI,^66^, ^69^ either because they develop later on or are more likely due to a bias as symptomatic children are more likely to be imaged. Cerebellar hypoplasia and dysplasia may affect the vermis (Figure 2), cerebellar hemispheres or both and are associated with a high risk of sequelae.^13^, ^66^ Focal WM anomalies and calcifications of deep nuclei can also be detected on fetal MRI (Figure 2), and their correct identification may require T1w or EPI images acquired in the coronal or sagittal planes.

**Figure 6:**
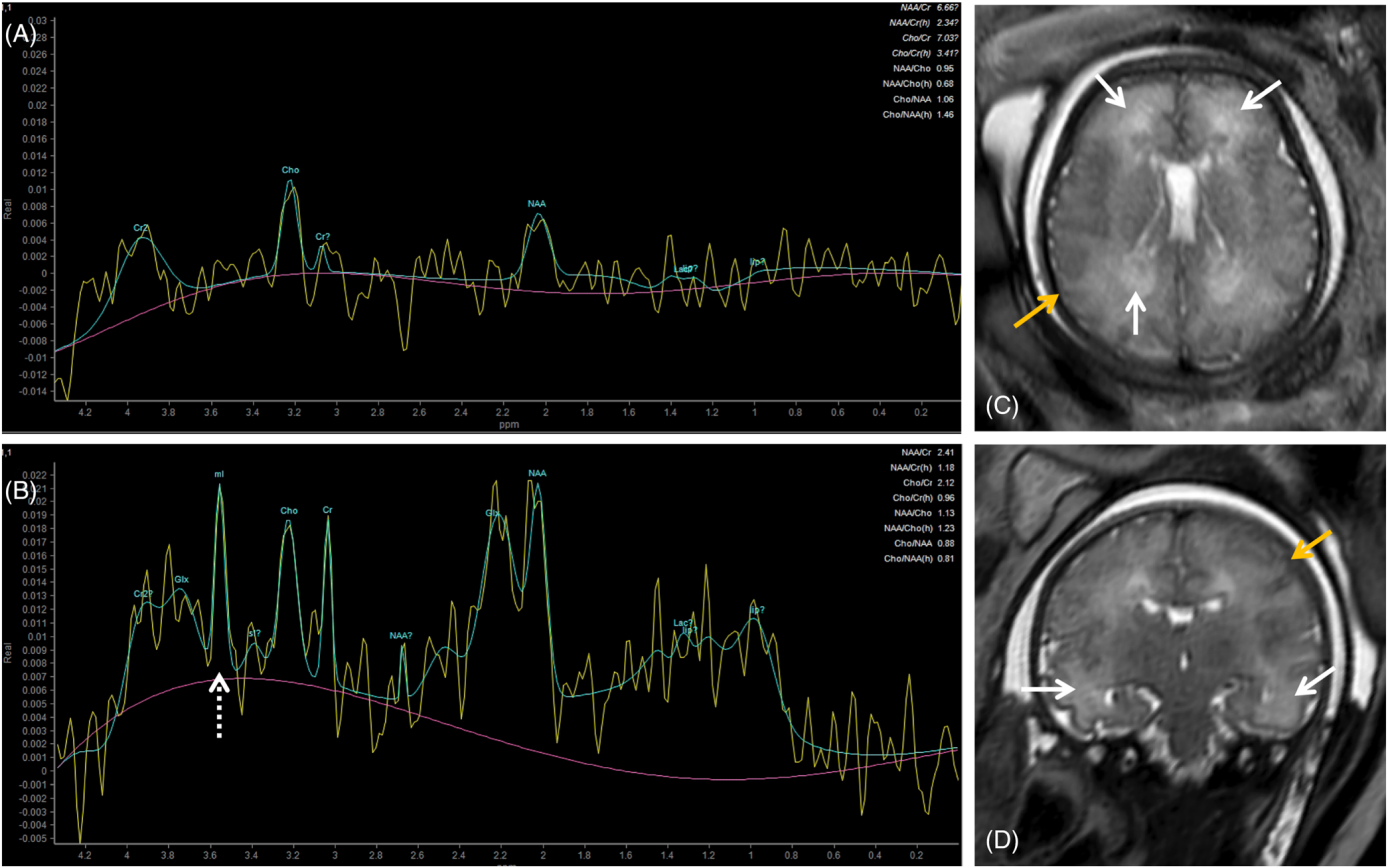
Comparison of intermediate (A, TE 144) and short (B, TE 31) TE spectroscopy of fetus referred for magnetic resonance imaging due to confirmed fetal cytomegalovirus infection and fetal growth restriction at 36 gestational weeks. T2 single shot fast spin echo images (C, axial; D, coronal) showed moderate brain swelling with extracerebral CSF spaces effacement (yellow arrows). There is white matter T2‐hyperintensity in the frontal, parietal‐occipital (C, white arrows) and to a lesser extent in the temporal lobe (D, white arrows). Magnetic resonance spectroscopy at TE 144 shows no significant changes; at TE 31 there is a myo‐inositol peak (B, white dashed arrow)

Advanced imaging techniques such as MR spectroscopy (MRS) and diffusion tensor imaging (DTI) have promising applications in cCMV. MRS allows assessment of the metabolic profiles of tissues in vivo, with an estimated success rate of 65%.^87^ In cCMV, MRS may show increased concentration of lipids, myo‐inositol (mI), and possibly alanine, in cases of T2w WM hyperintensities (Figure 6) and even normal‐appearing WM, which may correlate with viral brain infection.^88^ mI may also be a marker for increased astroglia/gliosis,^87^, ^88^ and is increased in neonates with hypoxic‐ischemic injuries^89^ which is one of the mechanisms through which the brain is thought to be involved in cCMV, secondary to placental involvement.^67^ mI has also been shown to be increased in fetal MRS in other longstanding insults such as congenital heart disease.^90^ Interpretation of MRS should take into consideration the gestational age, as mI and choline values decrease with advancing gestation while NAA and Cr resonances become more intense.^88^, ^91^, ^92^

DTI can also be useful in cCMV brain assessment. Tractography may help determine integrity of major WM tracts, particularly in cases of destructive lesions or in suspected WM anomalies, as can fractional anisotropy maps.^93^, ^94^

### 3.4 Extra‐CNS assessment

As neurodevelopmental sequelae are the main long‐term consequence of cCMV, fetal scans tend to focus on brain imaging. However, cCMV is a global process that involves not only the whole fetus, but also the placenta and AF.^51^, ^95^ Furthermore, given the lack of specificity of CNS imaging findings, adding fetal body information may help to improve diagnostic accuracy. A study by Marutama et al showed that abdominal findings were associated with a 21‐fold increase of death or neurological abnormality and combining abdominal and cerebral findings had the highest positive and negative predictive value (100%).^96^ There is however very limited literature on body fetal MRI in congenital CMV.

Fetal body anomalies associated with cCMV include hepatomegaly, splenomegaly, fetal growth restriction (FGR), hyperechogenic bowel, skin edema, ascites, pleural and pericardial effusion, hydrops, liver signal anomalies oligo‐/polyhydramnios, and placentomegaly.^73^, ^97^

**Figure 7:**
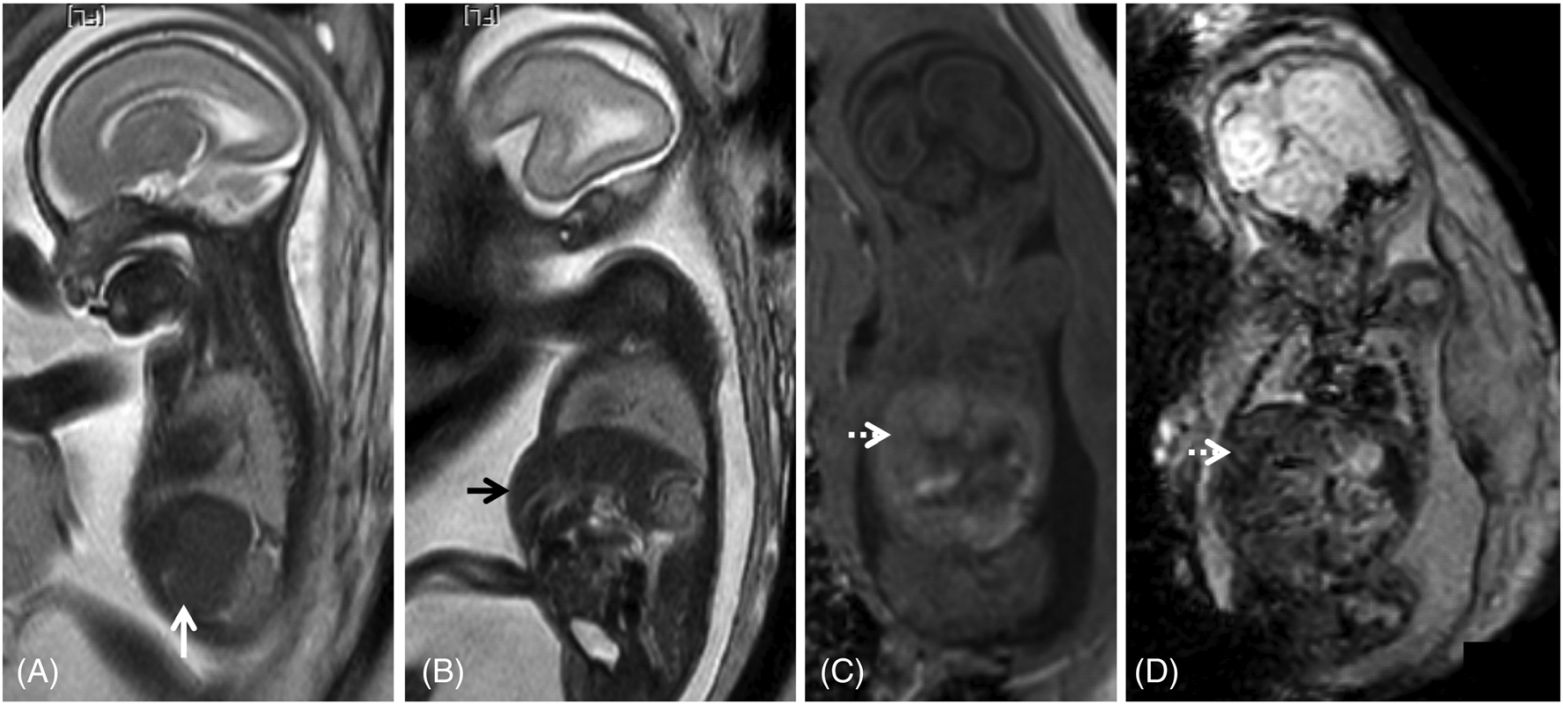
Fetal magnetic resonance imaging of a cytomegalovirus (CMV) positive fetus at 24 gestational weeks. T2WI depict splenomegaly (A, white arrow) and hepatomegaly (B, black arrow), protruding in the anterior abdominal wall. There is associated abnormal liver signal: isointense on T1WI (C, dashed white arrow) and on echo planar images (D, dashed white arrow), which cannot be identified on T2WI. These findings are often found in fetal infections and may help guide the diagnosis when brain anomalies are found, in the absence of a definite diagnosis of CMV infection

Hepato‐ and splenomegaly are common findings, readily identified both by US and fetal MRI (Figures 1 and 7).^65^ While focal liver calcifications are better identified on US, in whole liver involvement MRI is of major value showing low T1‐ and T2‐SI,^98^, ^99^ with our without high EPI SI, and may represent cirrhosis (Figure 7).^76^ Abnormal liver function testing at birth has been described in 7% of newborns, with a significant association with CNS sequelae (84%) and SNHL (54%).^48^

Echogenic bowel is a classical US sign of fetal infection although it may be overestimated.^100^, ^101^ It may be due to direct damage to the fetal intestine or as a result of other potential sequelae of congenital infection, such as ascites, intra‐amniotic bleeding or FGR. Isolated cases on US are associated with normal MRI and normal outcome^102^ as are grade 1 findings.^103^ If intra‐amniotic bleeding occurred, there may be increased T1w bowel signal, from fetal blood ingestion.

Abnormal fluid collections have been described in multiple compartments (pleural, pericardial, abdominal) and are easily visualized and characterized on MRI. MR signal is typically identical to CSF/AF on all sequences. T2w sequences are not sensitive to changes in fluid content; as such, T1w or FLAIR images may be useful to differentiate from proteinaceous effusions or chylothorax.^76^ Care should be taken when diagnosing pericardial effusions on T2w SSFP images, as myocardial contractions during acquisition may lead to false positives. Images should be compared to T2w single shot fast spin echo (ssFSE). They may cause secondary organ compression of the heart and lungs (Figure 5) with prognostic implications.^104^ Etiology may be multifactorial: anemia due to the combined effect of liver insufficiency and bone marrow infection, anoxia, endothelial cell damage, increased capillary permeability, and myocarditis.^13^, ^105^, ^106^ Nonimmune fetal hydrops may ensue in severe cases.

Cardiomyopathy is a rare finding. While cardiomegaly is readily identifiable on T2w ssFSE/SSFP images, for identification of the thickened myocardium specific cardiac gated images may be necessary. This may be a contributing factor to fetal hydrops and could be associated with tachyarrhythmia.^96^, ^106^

After reaching the fetal circulation, CMV preferentially involves the kidneys which may cause transient oligohydramnios and less frequently polyhydramnios,^13^, ^107^ although there are no specific descriptions of kidney signal anomalies on fetal MRI in this context.

FGR may develop as a result of either fetal or placental infection, or both.^65^ Further, FGR may be secondary to CMV placental involvement in the absence of fetal transmission.^13^, ^108^ Infants with FGR have a higher perinatal morbidity and mortality than infants with normal birth weights, as well as long term sequelae, and testing for cCMV may be warranted even if this is the sole finding.^109^ The placenta acts as a barrier against CMV but also as a reservoir, and poor outcome is also associated with viral replication, inflammation, edema, and fibrinoid development in the placenta.^109^ Placentitis, defined as a placental thickness of 4 cm or more, associated with a heterogeneous appearance for gestational age and possibly calcifications, is better identified on US.^73^ Although there is evidence that CMV‐related pathology is mediated by direct fetal infection, CMV infection of placental cells may also contribute to the pathogenesis of cCMV infection by altering placental formation and function, inducing placental damage and ultimately resulting in placental insufficiency.^67^ MR provides the advantage of allowing imaging of the whole placenta, as well as evaluation of placental function and reserve.^110^, ^111^

### 3.5 Imaging protocol

MRI protocols will vary between different centers, depending among other things on experience, availability of sequences, and findings during acquisition. T2w images should be obtained in the 3 planes for the fetal brain and body. T1w and diffusion weighted images should be obtained in at least one plane covering the fetus and placenta. Susceptibility sequences (EPI/T2*) help in detection of calcifications or hemorrhage.

Further sequences should be obtained as deemed appropriate. A run‐through of the sequences and their applicability in the context of CMV can be found in Table 2.

**Table 2:** Magnetic resonance imaging sequences and possible application in the context of congenital cytomegalovirus

	Application	
Sequence	Brain	Body/Placenta
T2w ssFSE	‐ Anatomical assessment ‐ Three orthogonal planes	‐ Anatomical assessment ‐ At least one plane
T2 SSFP	‐ Intraventricular lesion/septations	‐ Anatomical assessment ‐ Fetal heart/vessels ‐ Careful interpretation; if pericardial effusion suspected, confirm on T2 ssFSE
T1w	‐ Detection of hemorrhage and calcifications ‐ Bright periventricular rim in ventriculitis	‐ Detection of hemorrhage and calcifications ‐ Bowel assessment (meconium) ‐ Liver signal assessment
EPI/T2*	‐ Detection of hemorrhage and calcifications	‐ Detection of hemorrhage and calcifications ‐ Liver signal assessment
DWI/ADC	‐ Exclusion secondary lesions ‐ WM assessment	‐ Functioning kidney tissue detection
T2w—FLAIR	‐ Lamination ‐ WM assessment ‐ Intraventricular/periventricular lesions	‐ Fluid collection characterization (water/protein content)
T1w—FLAIR	‐ Myelination ‐ Calcifications ‐ Gliosis (?)	NA
DTI	‐ Tractography: reconstruction of WM fiber tracts. Assessment of presence or degree of disruption of major WM tracts in cases of destructive lesions. ‐ FA: assessment WM integrity	NA
MR spectroscopy (MRS)	‐ Metabolic profile brain tissue ‐ TE 35 ms: myo‐inositol, NAA, Choline ‐ TE 140 ms: AA, choline, Creatine, lactate	NA
CTG/Doppler US‐gated balanced SSFP	NA	‐ Evaluation of the fetal heart
Resting state functional MRI (fMRI) blood oxygenation level‐dependent contrast (BOLD)	‐ Assess functional connectivity (research)	‐ Placental imaging/placental reserve

Abbreviations: ADC, apparent diffusion coefficient; CTG, cardiotocogram; DTI, diffusion tensor imaging; DWI, diffusion weighted imaging; EPI, echo planar imaging; FA fractional anisotropy; FLAIR, fluid attenuation inversion recovery; MRS, Magnetic resonance spectroscopy; SSFP, steady state free precession; ssFSE, single shot Fast Spin Echo; WM, white matter.

### 3.6 Timing of imaging

Determining the ideal timing for imaging is not always straightforward. Although fetuses as young as 18 weeks can be successfully imaged, the physiological lissencephalic aspect of the brain at this point in development limits the ability to detect migration.^72^ Furthermore, enough time should have elapsed from infection to allow for structural changes to be detectable.

As demonstrated by Cannie et al, fetal MRI was equally reliable in predicting SNHL and neurological impairment at 27 and 33 gestational weeks (GW),^65^ challenging the general belief that imaging is more accurate later in pregnancy. In case of dubious or negative findings, or when parents or referring physicians need further information, a follow up examination may be helpful and reassuring. Moreover, a detailed third trimester fetal MRI may obviate the need for immediate postnatal MRI, avoiding newborn sedation.^72^

Another factor to be considered is the legal framework of termination of pregnancy that in many countries is limited to 24 weeks. Doneda et al have shown that detection of CMV‐associated brain anomalies is possible before this gestational age.^66^

### 3.7 Further considerations

Fetal MRI can be a useful tool in the assessment of cCMV. It can add information to US, for example, in 47% of cases at 25 weeks according to Doneda et al^66^ with follow up MR providing further information in 44.5%. Limitations of MRI have been highlighted by Birnbaum et al, with a higher false positive rate than US (17.5% [5% true FP and 12.5% inconclusive] vs 5% respectively).^63^ Notably, all false positive findings consisted of dubious WM hyperintensities (see below), and there was no postnatal imaging follow‐ups confirming or excluding these findings. The same authors noted that although MRI did not change counseling or outcome, it provided further reassurance for parents.^63^ It is also worth mentioning that although neurosonography provides a high NPV when the operator is aware of fetal infection (as high as 93%^73^), systematic ultrasound performed as part of routine antenatal care has a poor (as low as 35%) sensitivity.^112^

Beyond the discussion of what technique is superior, attaining an accurate diagnosis and as much prognostic information as possible is the ultimate goal for each patient. Decision on the need for fetal MRI should be made on a case‐to‐case basis and taking into consideration the objective or subjective need for further information or reassurance of referring physicians and parents.

In terms of MRI findings, anterior temporal lobe lesions are the most specific finding, and an area where MRI may add information to US. Other findings albeit unspecific should raise the suspicion of fetal infection, particularly if found in combination. This highlights the need for a full fetal assessment, as these findings may be outside the SNC.

Some authors have tried to stratify the risk of sequelae in relation to MRI findings. Leruez‐Ville et al stratified imaging findings in two categories (on US): mild brain anomalies (mild ventriculomegaly [<15 mm], intraventricular adhesions, calcifications, subependimal cysts, choroid plexus cysts) and severe brain anomalies (severe ventriculomegaly [>15 mm], periventriculitis, hydrocephalus, microcephaly, increased cisterna magna [>8 mm], porencephaly, lissencephaly, cystic lesions of the WM, and agenesis of the corpus callosum), to be evaluated together with the viral load.^73^

Connie et al suggested five grades of imaging findings using fetal MRI and postnatal outcomes, from mild to severe: normal, isolated frontal/parieto‐occipital white matter hyperintensity; temporal periventricular hyperintensity; temporal/occipital cysts and/or intraventricular septa; migration disorders, cerebellar hypoplasia and/or microcephaly.^65^ Despite not covering all possible findings, such as frontal cysts or calcifications, these findings correlated to neurodevelopmental outcome and SNHL postnatally.^65^

Although some of the features overlap, there is no single clear classification of findings that can be universally used currently, and more comprehensive studies integrating fetal MRI findings may be needed for a consensus to be reached.

However, some of the MRI findings are somewhat subjective. WM hyperintensities may be difficult to interpret, particularly in older fetuses when this finding can be normal. Other sequences, such as T2W FLAIR, DWI, or DTI can help determine their significance, as in normal subjects the WM signal changes tend to manifest exclusively on the T2WI. Additional sequences, such as spectroscopy can also be useful by showing mI or lactate peaks, suggestive of metabolic changes in the parenchyma, contributing to the added value of MRI. This is an example on how advanced fetal MRI may help to bridge the gap between lack of imaging findings and sequelae. It is a new and exciting field where further studies are necessary.

## 4 CONCLUSION

More awareness is needed regarding cCMV among parents, clinicians and radiologists. Given the paucity of unspecific symptoms and the lack of structured screening, many of these patients will not be diagnosed if the hypothesis of infection is not raised by findings on fetal imaging. This has severe consequences in treatment and patient counseling for present and future pregnancies. Anterior temporal lobe lesions are the most specific MR finding, and an area where MRI may add information to US. Other findings albeit unspecific should raise the suspicion of fetal infection, particularly when found in combination. This highlights the need for a full fetal assessment, as these findings may be outside the SNC. Advanced MR sequences may help in determining prognosis, but further studies are needed to assess their significance.

## CONFLICT OF INTEREST

The authors declare no conflicts of interest.

## ETHICAL STATEMENT

No ethics approval was obtained as there were no new data acquired for this review. All given data is cited in text and reference list which can be used for more information.

## Supporting information


**Figure S1** Example of normal fetal brain MRI at 35 GW in fetus referred to imaging for borderline ventriculomegaly. White matter heterogeneity can be identified on T2WI in the frontal (d, white dashed arrows) and parietal‐occipital parieto‐occipital (d, white arrows) regions. On T2w EPI‐FLAIR image (e) there is no corresponding hypointensity except in the expected gyral crests corresponding to remnants of the subplate (e, black dashed and full arrows).

**Figure S2** Further example of MRS in white matter hyperintensities in cCMV fetus at 33 GW. White matter heterogeneity can be identified on T2WI in the frontal (a, white dashed arrows) and parietal‐occipital parieto‐occipital (a, b, white arrows) regions. On T2w EPI‐FLAIR (c) there is no corresponding hypointensity except in the expected temporal pole region, corresponding to remnants of the subplate (c, blue arrow). On the MRS (TE 35 ms) there is an age appropriate spectrum, with no increased mI peak (d).

## Data Availability

Data sharing is not applicable to this article as no new data were created or analyzed in this study.
