# Supplementary figures and images for: The MRI spectrum of congenital cytomegalovirus infection
Source: Prenat Diagn. 2020 Jan 6;40(1):110–24. doi: 10.1002/pd.5591 (PMC7027449; doi:10.1002/pd.5591)

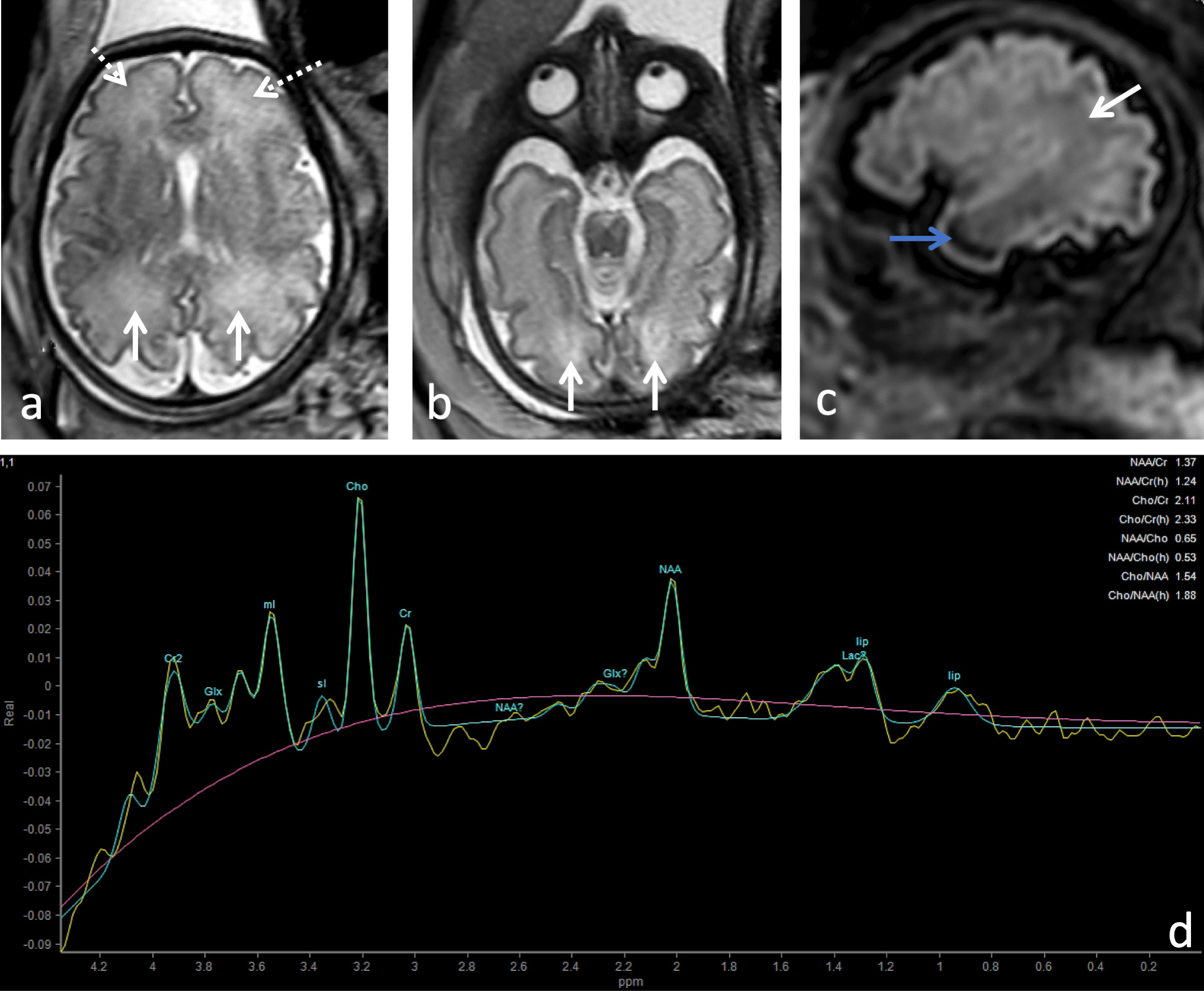

Supplement: Supplementary file 1 — Figure S1 Example of normal fetal brain MRI at 35 GW in fetus referred to imaging for borderline ventriculomegaly. White matter heterogeneity can be identified on T2WI in the frontal (d, white dashed arrows) and parietal‐occipital parieto‐occipital (d, white arrows) regions. On T2w EPI‐FLAIR image (e) there is no corresponding hypointensity except in the expected gyral crests corresponding to remnants of the subplate (e, black dashed and full arrows). [file PD-40-110-s001.tiff]

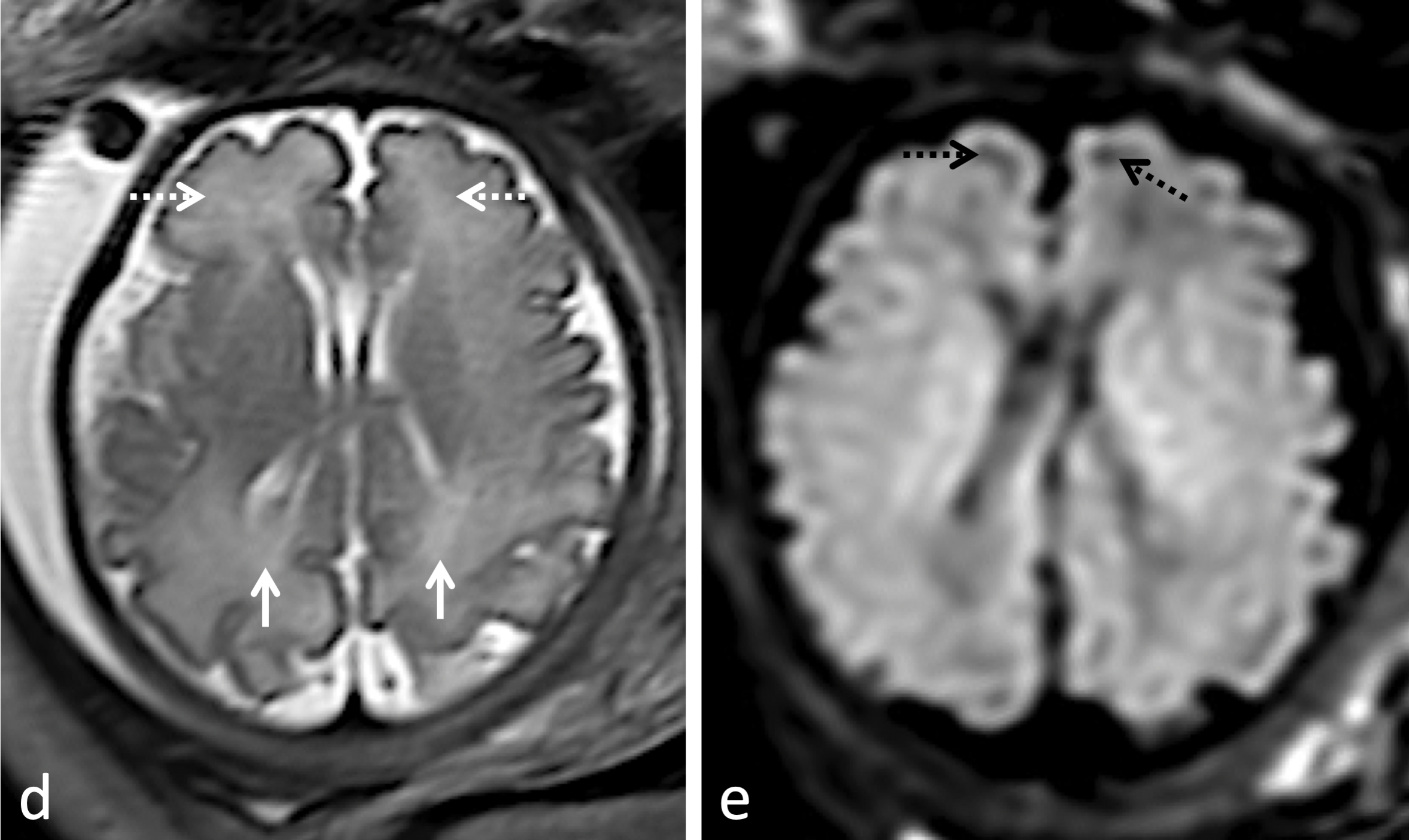

Supplement: Supplementary file 2 — Figure S2 Further example of MRS in white matter hyperintensities in cCMV fetus at 33 GW. White matter heterogeneity can be identified on T2WI in the frontal (a, white dashed arrows) and parietal‐occipital parieto‐occipital (a, b, white arrows) regions. On T2w EPI‐FLAIR (c) there is no corresponding hypointensity except in the expected temporal pole region, corresponding to remnants of the subplate (c, blue arrow). On the MRS (TE 35 ms) there is an age appropriate spectrum, with no increased mI peak (d). [file PD-40-110-s002.tiff]
